# Supplementary material for: Cis-regulatory variants affect gene expression dynamics in yeast
Source: eLife. 2021 Aug 9;10:e68469. doi: 10.7554/eLife.68469 (PMC8367379; doi:10.7554/eLife.68469)
Supplement: Transparent reporting form [file elife-68469-transrepform.pdf]

Westbrook Centre
Milton Road
Cambridge CB4 1YG, UK
P 01223 855340
W elifesciences.org
T @elife

eLife's transparent reporting form

We encourage authors to provide detailed information *within their submission* to facilitate the interpretation and replication of experiments. Authors can upload supporting documentation to indicate the use of appropriate reporting guidelines for health-related research (see EQUATOR Network), life science research (see the BioSharing Information Resource), or the ARRIVE guidelines for reporting work involving animal research. Where applicable, authors should refer to any relevant reporting standards documents in this form.

If you have any questions, please consult our Journal Policies and/or contact us: editorial@elifesciences.org.

Sample-size estimation

- You should state whether an appropriate sample size was computed when the study was being designed
- You should state the statistical method of sample size computation and any required assumptions
- If no explicit power analysis was used, you should describe how you decided what sample (replicate) size (number) to use

Please outline where this information can be found within the submission (e.g., sections or figure legends), or explain why this information doesn't apply to your submission:

RNA-seq gene expression dynamics: We used a sample size of five strains, three intra-specific hybrids and two inter-specific hybrids. As described in the results, we chose five hybrids to survey a range of strain divergence and because we did not know what level of divergence would be optimal for our study. Our rationale was that as divergence increases there should be more expression differences but also more sequence differences that could explain the expression divergence. Expression dynamics were independently assessed in each hybrid and so the power to detect them depends on the number of time-points rather than the number of strains. Gene expression was measured at 19 time-points during the diauxic shift. The number of time-points was based on the highest density of sampling during the diauxic shift. We chose 15 minute intervals since that is how long it takes to process each sample during the experiment.

CRE-seq expression dynamics: We used the same or more sampling time-points as the RNA-seq experiments so they would be comparable. As described in the methods, we used four replicate barcodes per CRE sequence. This was chosen to maximize the number of CRE sequences that could be tested while still being able to statistically assess the results.

Replicates

- You should report how often each experiment was performed
- You should include a definition of biological versus technical replication

eLife Sciences Publications, Ltd is a limited liability non-profit non-stock corporation incorporated in the State of Delaware, USA, with company number 5030732, and is registered in the UK with company number FC030576 and branch number BR015634 at the address Westbrook Centre, Milton Road, Cambridge CB4 1YG.

- The data obtained should be provided and sufficient information should be provided to indicate the number of independent biological and/or technical replicates
- If you encountered any outliers, you should describe how these were handled
- Criteria for exclusion/inclusion of data should be clearly stated
- High-throughput sequence data should be uploaded before submission, with a private link for reviewers provided (these are available from both GEO and ArrayExpress)

Please outline where this information can be found within the submission (e.g., sections or figure legends), or explain why this information doesn't apply to your submission:

Changes in gene expression over time were measured from samples of the same culture as described in the methods. There were 19 time-points to assess time as the independent variable. Because these time-points were taken from the same culture there is only one biological replicate and 19 technical replicates. However, the Durbin-Watson test is a valid measure of time-dependence as it tests the null hypothesis that error is serially uncorrelated. All technical samples (RNA extraction, library prep, sequencing) were completed independently and so there should be no serial correlation due to technical error. Yeast cultures can vary due to biological variation, e.g. growth rate, glucose consumption etc. However, this biological variation was accounted for because all comparisons were paired (two alleles) and experienced the same culture and even cellular environment. Similar to the RNA-seq experiments, we used the same approach and measured CRE-seq expression from a single culture of the pooled library, with all CREs experiencing the same environment.

Exclusion based on missing data is described in the methods section separately for the RNA-seq data and the CRE-seq data.

Sequencing data: Genome and RNA sequencing were deposited into NCBI's SRA and GEO database, respectively, as described in the data availability section. Barcode sequencing counts are available through the Open Science Framework portal as described in the data availability section.

eLife Sciences Publications, Ltd is a limited liability non-profit non-stock corporation incorporated in the State of Delaware, USA, with company number 5030732, and is registered in the UK with company number FC030576 and branch number BR015634 at the address Westbrook Centre, Milton Road, Cambridge CB4 1YG.

Statistical reporting

- Statistical analysis methods should be described and justified
- Raw data should be presented in figures whenever informative to do so (typically when N per group is less than 10)
- For each experiment, you should identify the statistical tests used, exact values of N, definitions of center, methods of multiple test correction, and dispersion and precision measures (e.g., mean, median, SD, SEM, confidence intervals; and, for the major substantive results, a measure of effect size (e.g., Pearson's r, Cohen's d)
- Report exact p-values wherever possible alongside the summary statistics and 95% confidence intervals. These should be reported for all key questions and not only when the p-value is less than 0.05.

Please outline where this information can be found within the submission (e.g., sections or figure legends), or explain why this information doesn't apply to your submission:

ASE levels and dynamics: Sample sizes, statistical tests and multiple test correction are described in the methods section. Significance for tests of ASE levels and ASE dynamics for each gene are in ASE.stats.csv.

Logistic regression: The sample size, logistic regression and multiple test correction used to test for associations between ASE and SNPs/InDels is described in the methods section. The effect size, confidence interval and p-values are in Table S4, Figure 2 and Figure2_data.csv.

CRE-seq expression levels and dynamics: Sample sizes, statistical tests and multiple test correction are described in the methods section. Significance for each tested CRE is in intra.stats.csv and inter.stats.csv for the intra-specific and inter-specific libraries, respectively.

All files mentioned here as well as all data underlying the figures and tables are available through OSF as described in the data availability section.

(For large datasets, or papers with a very large number of statistical tests, you may upload a single table file with tests, Ns, etc., with reference to sections in the manuscript.)

Group allocation

- Indicate how samples were allocated into experimental groups (in the case of clinical studies, please specify allocation to treatment method); if randomization was used, please also state if restricted randomization was applied
- Indicate if masking was used during group allocation, data collection and/or data analysis

Please outline where this information can be found within the submission (e.g., sections or figure legends), or explain why this information doesn't apply to your submission:

Not applicable.

eLife Sciences Publications, Ltd is a limited liability non-profit non-stock corporation incorporated in the State of Delaware, USA, with company number 5030732, and is registered in the UK with company number FC030576 and branch number BR015634 at the address Westbrook Centre, Milton Road, Cambridge CB4 1YG.

Additional data files ("source data")

- We encourage you to upload relevant additional data files, such as numerical data that are represented as a graph in a figure, or as a summary table
- Where provided, these should be in the most useful format, and they can be uploaded as "Source data" files linked to a main figure or table
- Include model definition files including the full list of parameters used
- Include code used for data analysis (e.g., R, MatLab)
- Avoid stating that data files are "available upon request"

Please indicate the figures or tables for which source data files have been provided:

Analysis scripts, data and files underlying figures are available through the Open Science Framework as described in the data availability section.

eLife Sciences Publications, Ltd is a limited liability non-profit non-stock corporation incorporated in the State of Delaware, USA, with company number 5030732, and is registered in the UK with company number FC030576 and branch number BR015634 at the address Westbrook Centre, Milton Road, Cambridge CB4 1YG.
